# Sylvian Fissure Meningiomas: Case Report and Literature Review

**DOI:** 10.3389/fonc.2020.00427

**Published:** 2020-04-16

**Authors:** Chengwei Cai, Zhoule Zhu, Xinxia Guo, Xiaoming Guo, Hongjie Jiang, Zhe Zheng, Jianmin Zhang, Anwen Shao, Junming Zhu

**Affiliations:** Department of Neurosurgery, The Second Affiliated Hospital of Zhejiang University School of Medicine, Hangzhou, China

**Keywords:** meningiomas, Sylvian fissure, case report, atypical, neurosurgery

## Abstract

Meningiomas are primary intracranial tumors derived from arachnoid cap cells or meningothelial cells and usually display dural attachment. However, a small proportion of meningiomas that arise from the Sylvian fissure do not manifest dural attachment. Sylvian fissure meningiomas are relatively rare and have differential characteristics as compared with typical meningiomas. Herein, we reported a special case of atypical meningioma in the Sylvian fissure, that showed non-enhancement after contract management. The patient was a 30-year-old woman with a 2-year history of seizures. Preoperative computerized tomography and magnetic resonance imaging scans showed a calcific, non-enhancing lesion in the right insula lobe. The lesion was excised surgically for seizure control. Intraoperatively, the tumor was observed to be closely adhered to the middle cerebral artery (MCA), resulting in mild arterial damage. A case of Sylvian fissure meningioma was ultimately diagnosed by histopathological examination of the resected specimens. Sylvian fissure meningiomas are closely associated with the MCA and exhibit unusual imaging characteristics. Preoperative misdiagnosis may have serious adverse consequences and may result in incorrect surgery. To improve awareness of Sylvian fissure meningiomas on the differential diagnosis of Sylvian fissure lesions among clinicians, we present this report and briefly summarize previously reported cases to describe the clinical, pathological, radiological, and surgical features.

## Introduction

Generally, meningiomas originate from the dura mater; however, some cases originate from the Sylvian fissure and show non-dural attachment. These cases can be easily missed preoperatively or may be misdiagnosed. Atypical meningioma is a distinct meningioma subtype and accounts for a small proportion of meningiomas with poor prognosis (1). At present, 38 cases of Sylvian fissure meningiomas have been reported (2–32), and four cases presented an atypical type (19, 22, 25, 30), but none were non-enhanced lesions (30). In this report, we describe a patient who presented with an intracranial mass in the right insula lobe, which was clinically diagnosed as a low-grade glioma based on the results of preoperative neuroimaging, with a subsequent revised diagnosis of meningioma confirmed by postoperative histopathology. In contrast with previous reported cases, our case presented characteristic non-enhancement of the meningioma lesion on contrast-enhanced magnetic resonance imaging (MRI), which is unique. In addition, we have reviewed the relevant literature and summarized the major findings herein to improve the awareness of Sylvian fissure meningiomas among clinicians for the differential diagnosis of Sylvian fissure lesions.

## Case Presentation

**Figure 1:**
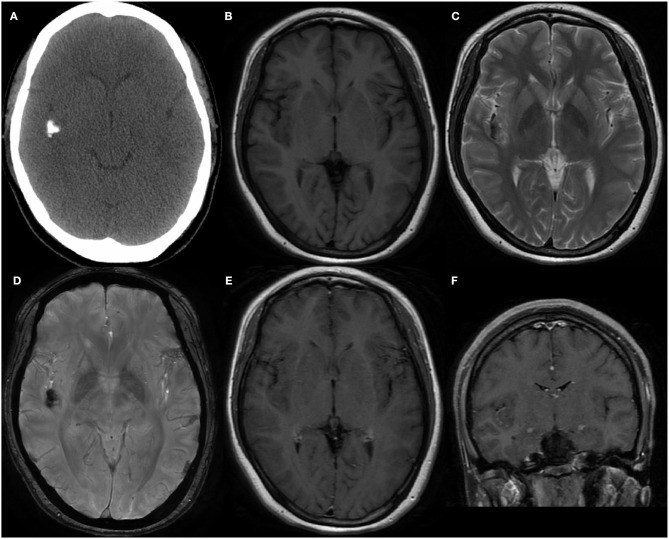
**(A)** The Computed tomography demonstrated the calcification in right Sylvian fissure and posterior part of insula. **(B,C)** MRI scans revealed a lesion without dural attachment located in right insular lobe which was mainly hypointense on both T1-weighted and T2-weighted. **(D)** Lesion showed hypointense on susceptibility weighted imaging. Enhanced MRI showed no obvious enhancement after gadolinium administration in axial **(E)** and coronal **(F)** position.

The patient was a 30-year-old homemaker with a 2-year history of seizures before admission. She had previously been treated with a routine antiepileptic (sodium valproate; 1,000 mg per day) for 3 months; however, the drug was weaned off because of its side effect (weight gain). Subsequently, she was prescribed lamotrigine (200 mg per day) and topiramate (75 mg per day) for seizure control, but continued to have occasional seizures, once or twice a month. She had no other neurological deficit or past medical history, and results of systemic examinations were normal. She had no family history of hereditary diseases. Electroencephalography performed after admission detected epileptiform activity in the right cerebral hemisphere. Computerized tomography (CT) demonstrated a calcific lesion in the right Sylvian fissure and posterior part of the insula (Figure 1A). An MRI scan revealed a lesion in the right insular lobe without any dural attachment, which was primarily hypointense on both T1- and T2-weighted MRI (Figures 1B,C). Additionally, the lesion appeared hypointense on susceptibility-weighted imaging (Figure 1D), with no obvious enhancement despite administration of gadolinium contrast (Figures 1E, F). The results of all preoperative laboratory tests were within normal ranges. Preoperatively, low-grade glioma, including oligodendroglioma, and diffuse astrocytoma, was suspected.

**Figure 2:**
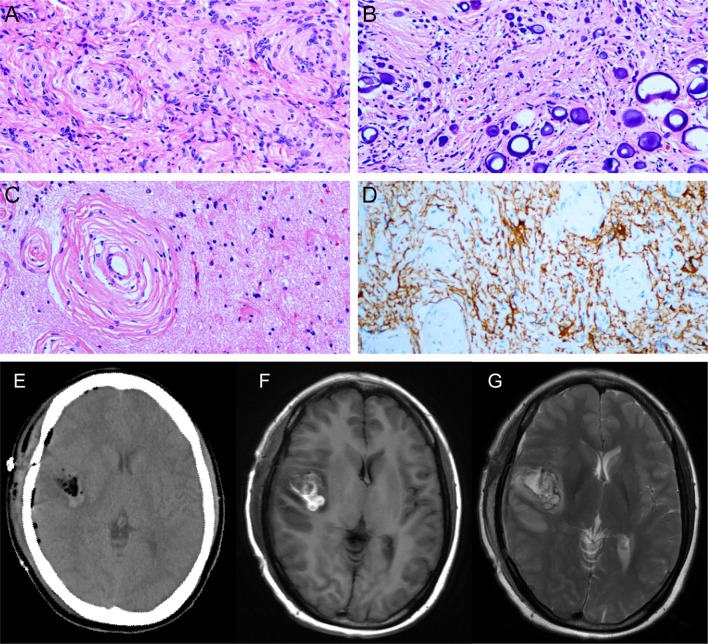
**(A)** The tumor tissue arranged in swirl structure to form the meningeal corpuscle. **(B)** Psammoma bodies. **(C)** The meningeal corpuscles invade normal brain tissue. **(D)** GFAP staining show brain tissue (+) and meningeal corpuscles (-). Postoperative CT **(E)** and MRI **(F,G)** showed tumor gross total resection.

With this provisional diagnosis, the patient was referred for surgical management and underwent a right temporal craniotomy for gross total resection of the tumor and for seizure control. Upon opening the dura and separating the Sylvian fissure, a gray-white lesion (diameter, about 25^*^20 mm) was observed. It had a relatively elastic consistency and the mass partially invading the brain parenchyma of the insula, was densely adhered to the branches of the medial cerebral artery (MCA), and had partial calcification. Intraoperatively, despite careful micro-dissection, the MCA perforators were mildly damaged. Ultimately, several surgical specimens of the excised tumor were sent for histopathological analysis. Postoperatively, the results of histopathological examinations revealed a World Health Organization (WHO) grade II meningioma of atypical type (Figures 2A–D). Immunohistochemical examinations revealed negative immunoreactivity for Ki-67, progesterone receptor, p53, epithelial membrane antigen, and positive immunoreactivity for vimentin, and somatostatin receptor 2.

Postoperatively, the patient regained consciousness with a mild clinical symptom of hemiplegia in her left limbs, which lasted for nearly 2 weeks but improved gradually with rehabilitation therapy. A follow-up gadolinium-enhanced MRI (postoperative 2 weeks) showed complete total excision of the tumor (Figures 2E–G). The patient presented no other postoperative neurological deficit or seizure recurrence for approximately a year and a half. An MRI scan will be performed annually to continuously monitor for any evidence of tumor recurrence.

## Discussion

**Figure 3:**
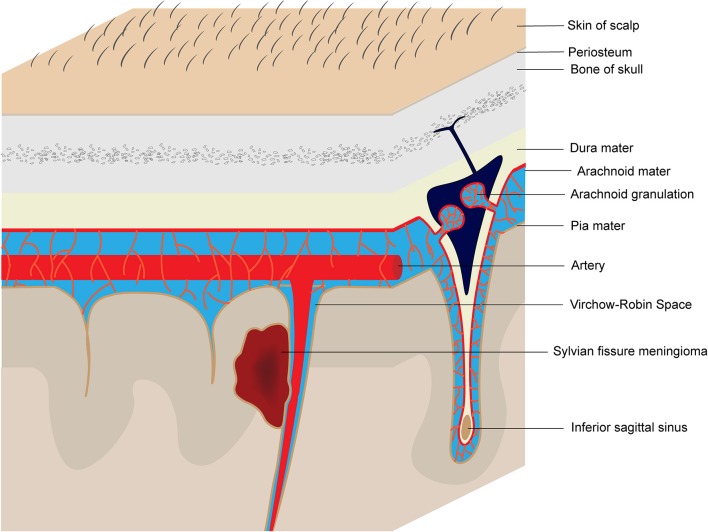
Schematic diagram of the origin of Sylvian fissure meningiomas.

Meningiomas are the second most common primary central nervous system tumors (CNST), accounting for approximately one-third of the primary CNST cases (33, 34). They are postulated to originate from the arachnoid cap and meningothelial cells, which are present in the arachnoid layer of the meninges or Pacchionian granulation, and typically display dural attachment. Occasionally, the arachnoid cap cells can appear in the pia mater of the Sylvian fissure or Virchow-Robin space along the MCA or its branches (23, 31) (Figure 3). Our patient presents an additional case of Sylvian fissure meningioma with unusual imaging characteristics and a rare atypical subtype.

The mean onset age of typical meningiomas is about 55 years, and the incidence increases with age, with women having a higher morbidity than men (34). However, the clinical characteristics of Sylvian fissure meningiomas are different. A retrospective analysis of 38 [25 men (65.8%) and 13 female (34.2%)] patients with Sylvian fissure meningiomas reported from 1938 to 2019 (Table 1) revealed that the average onset age is about 22.5 ± 17.4 years. Seizure was the major symptom of Sylvian fissure meningiomas (65.8%, 25 patients). Other reported symptoms included headache (39.5%, 15 patients) and hemiparesis (18.4%, 7 patients). Rare instance of initial symptom included visual impairment (2.6%, 1 patient), incidental (2.6%, 1 patient), and dizziness (2.6%, 1 patient). The incidence of seizures in patients with Sylvian fissure meningiomas (65.8%) was reported to be higher than those with common supratentorial meningiomas (29.2%) (35). Generally, seizure frequency depends on tumor location, and the limbic and temporal lobe had the lowest threshold for producing a seizure. Also, hypoxia and metabolic imbalances caused by tumor invasion and extrusion were the potential etiological mechanisms (36).

**Table 1:** Summary of Sylvian fissure meningiomas up to October 31, 2019.

**References**	**Age/Sex**	**Clinical features**	**Imaging features**	**Removal**	**Histopathology**	**Follow-up**
Cushing et al. (2)	8/M	Epilepsy	N/A	Subtotal	Psammomatous	5y: died
	48/F	Epilepsy	N/A	Subtotal	Psammomatous	1d: died
Barcia-Goyanes et al. (3)	20/F	Epilepsy	N/A	N/A	Psammomatous	N/A
Mori et al. (4)	23/M	Epilepsy	N/A	Subtotal	Transitional	N/A
Saito et al. (5)	31/F	Epilepsy	Hyperdense lesion in CT	Gross total	Psammomatous	N/A
Tsuchida et al. (6)	46/M	Headache	N/A	Gross total	Psammomatous	4y: relapse-free
Awa et al. (7)	16/M	Headache	N/A	Gross total	Meningothelial	2y: relapse-free
Okamoto et al. (9)	27/F	Headache	N/A	Gross total	Fibroblastic	5y: died
	35/F	Headache and visual impaired	Severe edema, hyperdense, and homogeneous lesion in CT	Gross total	Fibroblastic	N/A
Drake et al. (8)	3/F	Headache	N/A	Gross total	Malignant	N/A
Hirao et al. (10)	34/F	Epilepsy	Homogeneous enhancement in CT	Gross total	Fibroblastic	N/A
Silbergeld et al. (11)	4/F	Epilepsy	Homogeneous enhancement in CT	Subtotal, radiation therapy	Meningothelial	N/A
Cho et al. (12)	2/M	Epilepsy and hemiparesis	Severe edema, hyperdense with homogeneous lesion in CT	Gross total	Transitional	2y: relapse-free
Graziani et al. (13)	19/M	Headache and hemiparesis	Moderate edema, calcifications, Hypointense in T1 and T2	Gross total	Psammomatous	N/A
Mori et al. (15)	12/M	Headache	Slight edema, well-enhanced tumor in CT and MRI	Gross total	Transitional	1y: relapse-free
Chiocca et al. (14)	26/F	Epilepsy	Slight edema, hypointense and homogeneous in T1 and T2	Gross total	Fibroblastic	N/A
Matsumoto et al. (16)	62/F	Epilepsy	Hypointense in T1 and T2	Gross total	Psammomatous	N/A
Cooper et al. (17)	4/M	Headache	Severe edema, heterogeneous intensity in T1 and T2	Gross total	Transitional	1y: relapse-free
Mitsuyama et al. (18)	1/M	Epilepsy	Well-enhanced tumor in CT and MRI	Gross total	Fibroblastic	N/A
Kaplan et al. (19)	11/M	Epilepsy	Isointense in T1 and heterogeneous intensity in T2	Gross total	Atypical	N/A
Chang et al. (20)	35/M	Epilepsy	Severe edema, isointense, and homogeneous in T1 and T2	Subtotal, gamma-knife	Transitional	N/A
Mclver et al. (21)	23/M	Epilepsy	Heterogeneous intensity in T1 and T2	Subtotal	Chordoid	17m: stable residual tumor
Samson et al. (23)	6/M	Epilepsy	Calcification, edema, heterogeneous enhancement, hypointense on T1 and hyperintense on T2	Gross total	N/A	4y: relapse-free
Cecchi et al. (22)	23/M	Headache and hemiparesis	Moderate edema, heterogeneous intensity in T1 and T2	Subtotal, radiation therapy	Atypical	2y: stable residual tumor
Ma et al. (25)	53/M	Epilepsy	Homogeneous enhancement, edema	Subtotal, gamma-knife	Atypical	2y: stable residual tumor
Chae et al. (24)	69/M	Incidental	Calcification, edema, heterogeneous intensity in T2, heterogenous enhancement	Subtotal	Psammomatous	N/A
Aras et al. (26)	15/M	Epilepsy	Homogeneous enhancement, hypointense on T1 and iso-hypointense on T2, mild edema	1st surgery: subtotal 2nd surgery: gross total	Fibroblastic	3y: relapse-free
	28/M	Epilepsy and hemiparesis	Heterogenous enhancement, hypointense on T1 and heterogeneous intensity on T2, edema	1st surgery: subtotal 2nd surgery: gross total	Meningothelial	5y: relapse-free
Kim et al. (27)	43/M	Epilepsy	Minimal calcification, Isointense in T1 and T2, ring like enhancement, edema	Subtotal	Lymphoplasmacyte-rich	4y: stable residual tumor
Fukushima et al. (28)	10/M	Epilepsy	Heterogeneous enhancement	Subtotal	Sclerosing	1y: stable residual tumor
Donovan et al. (29)	11/M	Epilepsy	Calcification, homogenous enhancement	Gross total	Transitional	10y: relapse-free
	7/M	Epilepsy and headache	Calcification, homogenous enhancement	1st surgery: subtotal 2nd surgery: subtotal	Fibroblastic	2y: stable residual tumor
	16/F	Epilepsy	Partial calcification, minimal enhancement	Subtotal	Meningothelial	5y: stable residual tumor
Brogna et al. (30)	32/M	Headache and dizziness	Isointense on T1 and T2, homogenous enhancement	Gross total	Atypical	3y: relapse-free
Yamagishi et al. (32)	32/M	Headache	Isointense on T1 and T2, homogenous enhancement	Gross total	Transitional	6m: relapse-free
Amirjamshidi et al. (31)	7/F	Headache and hemiparesis	Calcification, edema, isointense on T1 and T2, vivid enhancement	Gross total	Meningothelial	13y: relapse-free
	5/F	Headache, epilepsy, and hemiparesis	Isointense on T1 and T2, homogenous enhancement	Gross total	Meningothelial	5y: relapse-free
	7/M	Headache, epilepsy, and hemiparesis	Isointense on T1, homogenous enhancement	Gross total	Meningothelial	2y: relapse-free

The majority of common meningiomas are WHO grade I, with approximately 16.9% cases being atypical (WHO grade II) (37). However, the proportion is expected to rise according to the newly recommended WHO 2016 criteria for the classification of atypical meningioma (38). Sylvian fissure meningiomas comprise three types of WHO grades, and previously reported cases were histologically diagnosed as psammomatous (8 cases, 21.1%), transitional (7 cases, 18.4%), meningothelial (7 cases, 18.4%), fibroblastic (7 cases, 18.4%), atypical (4 cases, 10.5%), chordoid (1 case, 2.6%), sclerosing (1 case, 2.6%), lymphoplasmacyte-rich (1 case, 2.6%), and malignant (1 case, 2.6%). The arachnoid cells in all of these cases can manifest divergent differentiation. Presence of brain invasion, which was added to the histological criteria, alone can aid the diagnosis of atypical meningiomas according to the 2016 WHO classification of CNSTs (38). Invasion of the brain parenchyma in our case confirmed a final diagnosis of atypical type meningioma with WHO II grade, which is the fifth case reported till date.

The main radiological characteristics of meningiomas generally include extra-axial occupation and dura mater attachment (dural tail sign). Contrarily, radiological imaging features of Sylvian fissure meningiomas have differential characteristics, such as intra-axial mass without dural attachment. It is extremely difficult to differentiate Sylvian fissure meningioma from other intracranial masses, such as low-grade glioma, teratoma, metastasis, cavernous hemangioma, tuberculous granuloma, etc. The previously reported 38 cases of Sylvian fissure meningiomas frequently showed hypointense or isointense lesions on T1- and T2-weighted MRI, with homogeneous or heterogeneous enhancement after contrast administration. Some cases also showed calcification or edema on CT and MRI scans, but these features were non-specific. Interestingly, the tumor in our case presented obvious calcification with negligible enhancement on the preoperative contrast-enhanced MRI scan, which significantly complicated the preoperative diagnosis. Meningiomas without enhancement have rarely been reported previously. Kubota et al. (39) and Zhang et al. (40) reported two cases of non-enhancement meningioma, due to the cystic or necrotic changes and distinctive pathological features. We considered that the tumor calcification, as a large number of psammoma bodies are found in pathology, result in non-enhancement in our case.

It is well-acknowledged that the progressive growth and enlargement of typical meningiomas can oppress or enclose the arteries. However, owing to the layer of arachnoid membrane between the blood vessels and tumors, it is relatively easy to intraoperatively discern and separate the meningiomas from the adjacent arteries. Because of the close relationship between the Sylvian fissure meningiomas with the MCA and its branches, blood vessels are more likely to be damaged during resection surgeries. In our case, a branch of the MCA was injured intraoperatively despite careful micro-dissection, and the patient had a mild cerebral infarction postoperatively. Fortunately, the patient recovered without any obvious neurological impairment after an adequate recovery period and remained seizure free for about a year and a half postoperatively. According to the previously reported cases, 22 (57.9%) patients achieved gross total resection in the first attempt and showed favorable outcomes with a relapse-free status (92.3%, 12/13 patients, some patients had no follow-up data) at follow-up even after several years. Of the 15 patients who underwent subtotal resection of tumor, three patients underwent a secondary surgery, and four patients accepted subsequent radiation or gamma-knife therapy. However, the residual tumors in all 15 patients showed no sign of further progression. Patients at risk of serious postoperative complications with gross total excision surgery can be recommended for radiation or gamma-knife therapy (11, 20, 22, 25). Atypical meningiomas are intermediate-grade tumor with a relatively greater risk of recurrence, requiring longitudinal monitoring by sequential radiological imaging. Optionally, radiation therapy can be used if required. The patient was satisfied with the overall treatment course, operation, and intensive nursing; moreover, her seizures are now well under control.

## Conclusion

Sylvian fissure meningiomas are rare, and a preoperative diagnosis is difficult without adequate knowledge of the case. Due to the special origin, Sylvian fissure meningiomas generally present non-dural attachment and have a close relationship with the MCA (30). Tumor onset usually occurs at a young age and seizures are the most common initial symptom. Sylvian fissure meningiomas have multiple pathological types and effective treatment can ensure a favorable prognosis. However, the tumor should be carefully resected in cases with arterial adherence to avoid collateral artery injuries and postoperative infarction. This study is the fifth report of a rare case of an atypical Sylvian fissure meningioma with unusual presentations on preoperative radiological examination. The case report adds new knowledge to the existing literature and will help to remind clinicians of the rare presentations of atypical meningioma.

## Ethics Statement

The studies involving human participants were reviewed and approved by Institutional Review Board of the Second Affiliated Hospital of Zhejiang University School of Medicine [Hangzhou]. Written informed consent was obtained from the patient for the publication of this case report.

## Author Contributions

CC and ZZhu: drafted the manuscript. XinG and XiaG: acquisition of data. HJ, ZZhe, and AS: analysis or interpretation of data. JZhu and JZha: study concept and design.

### Conflict of Interest

The authors declare that the research was conducted in the absence of any commercial or financial relationships that could be construed as a potential conflict of interest.
